# Early recognition of child abuse through screening indicators at the emergency department: experience of a tertiary urban pediatric hospital

**DOI:** 10.1186/s13052-022-01214-9

**Published:** 2022-02-18

**Authors:** Caterina Offidani, Alberto Villani, Antonino Reale, Maria Rosaria Marchili, Lelia Rotondi Aufiero, Patrizio Moras, Maria Lodise, Umberto Raucci, Maria Antonietta Barbieri, Massimiliano Raponi

**Affiliations:** 1 Unit of Legal Medicine, Bambino Gesù Children’s Hospital, IRCCS, Rome, Italy; 2 Department of Emergency, Acceptance and General Pediatrics, Bambino Gesù Children’s Hospital, IRCCS, Rome, Italy; 3 University of Rome Tor Vergata, Residency School of Pediatrics, Rome, Italy; 4 Academic Department of Pediatrics (DPUO), Bambino Gesù Children’s Hospital, IRCCS, Rome, Italy; 5 Medical Direction, Bambino Gesù Children’s Hospital, IRCCS, Rome, Italy

**Keywords:** Child abuse, Neglect, Maltreatment, Sexual abuse, Screening

## Abstract

**Background:**

Emergency Departments play a pivotal role in detecting cases of child abuse. Despite the efforts made in the past decades on the need for a screening method for the early detection of abuse victims, a unique instrument shared by the international scientific community has not been made. These instruments should be able to help recognizing whether it is necessary to further investigate the child’s condition. The aim of the study is to illustrate the screening indicators in use since 2010 in the Emergency Department of the Bambino Gesù Children’s Hospital to early recognise the victims of abuse and the modifying process of the screening tool undertaken over the years.

**Methods:**

We retrospectively analyzed the process that led to the editing of the indicators of child abuse in use nowadays at the Bambino Gesù Children's Hospital. We codified three clinical pathways to apply in case of suspected abuse. Furthermore, we investigated the medical records of screening-positive accesses in the Paediatric Emergency Department of the Bambino Gesù Children's Hospital from January 2008 to October 2020.

**Results:**

An estimation of positive screening, regarding the type of abuse suspected, and the number of accessed in ED was made, resulting in a cohort of 956 patients. In 2010 we created a list of 14 items grouped in three clusters: anamnestic declarations or incongruences, carelessness/neglect and evident lesions at physical examination. Positivity to one of the items allows the actuation of the investigating protocol named as clinical pathway.In 2013, after three years of experience, the criteria were edited to increase specificity. The application of screening led to a median number of 82 suspected cases/year from 2013 to 2020.

**Conclusion:**

A screening tool is essential and productive for the early recognition of victims of abuse. An in-deep analysis of suspected cases through a standardized method, such as the clinical pathway, allowed reaching the diagnosis in a more accurate and precise manner.

## Introduction

Child maltreatment and abuse are important social and medical problems. Victims of abuse are susceptible of dangerous short and long-term adverse consequences, such as mental and medical health issues [1–4]. Abuse is defined as a commissive or omissive act that can lead to physical or mental injury. It recognized as abuse a harmful behaviour, defined as one of the following: Neglect or Carelessness, Physical or Psychological maltreatment and Sexual Abuse. The true extent of child abuse and neglect (CAN) remains unknown and many published studies have been criticized for under-representation [5–10]. However, studies based on reporting by professionals or on administrative data performed in the US, Canada and the Netherlands show a national incidence rate of 1.6–3%. Given the numbers of children affected by child maltreatment and the direct consequences that can develop, prompt identification of child maltreatment is crucial [11–13].

Emergency Departments (ED) play a pivotal role in identifying child maltreatment as ED may represent a frequent entrypoint to health care for patients. Therefore, healthcare personnel in emergency setting may be the first hospital contact and opportunity for recognising CAN victims.

It is estimated that 2% to 10% of children visiting the ED are victims of CAN [14–16]. ED then represents a reception area for victims of abuse, but it is easy to understand that due the nature of the structure, dedicated to the urgencies and emergencies, overworked and characterized by the need for speed of decision-making, it could sometimes be difficult the prompt recognition of the child abused. The last National Institute for Health and Care Excellence (NICE) Clinical Guideline provides a detailed summary of alerting features, including physical injuries, to raise awareness and help healthcare professionals who are not specialists in child protection to identify children who may have been maltreated [17].

In ED are required easy-to-perform screening tools to recognize the suspected abuse condition, that healthcare professionals can easily use when historying patient data at triage. The use of this tools should be able to implement the further diagnostic pathway more suitable and tailored to the individual patient.

Screening methods are used in the ED to improve the identification of children with suspected CAN, which leads to a comprehensive assessment, termed as “clinical pathway”. The final purpose is to ascertain the cause of the injury, the type of abuse, the preventive and curative measures that could be done to ameliorate the condition of the children and, if necessary, their protection.

Despite the efforts made in the past decades on the need for a screening method for the early detection of abuse victims, a unique instrument shared by the international scientific community has not been made [18]. The aim of the study was to illustrate the screening indicators in use since 2010 in ED of the Bambino Gesù Children’s Hospital, an urban tertiary hospital in Rome, to early recognize the victims of abuse, and to describe the modifying process of the screening tool undertaken over the years. Additional aim was to show the clinical pathways applied on suspected patients by a multidisciplinary team to assess the risk of CAN.

## Methods

The study retrospectively analysed the process that led to the editing of the indicators of abuse in use nowadays at the Bambino Gesù Children's Hospital; we also codified three clinical pathways to apply in case of suspected abuse. We further investigated the medical records of screening-positive accesses in the Paediatric ED of the Bambino Gesù Children's Hospital from January 2008 to October 2020. In our Hospital, before the introduction of the screening method, the recognition of victims of abuse was solely based on the expertise of the ED physician personal background. The benefit of this method was the convenient simplicity of having a rapid and dependable experienced opinion when needed; the disadvantage consisted on basing the suspect on a single opinion, instead of a multidisciplinary assessment, other than a less stratified and verified methodology. After a process of revision on the results obtained by this approach, in 2009 the hospital administration decided to improve the detection methodology introducing the screening indicators. The clinical pathways, composed of 14 items, were firstly used in clinical practice from the 17^th^ of June 2010 The items tried to meet these crucial conditions: rapid execution, easy performance, realizable by all healthcare workers, with wide sensitivity and specificity. The screening method for early recognition of abuse has been applied systematically to all children entering ED. The screening is also applied in case of hospitalization in every other department of the hospital and at every visit. The items could be grouped in three clusters: anamnestic declarations or incongruences, carelessness/neglect and evident lesions at physical examination. All items are reported in Table 1. The first cluster underlines the importance of any declaration of abuse, domestic violence, and sexual abuse. Any suspicion of abuse originated in the healthcare worker that compiles the screening tool (nurses, doctors) is considered as one of the items. The second cluster focuses on the aspects of neglect or carelessness, also in terms of child exposure to drugs, venoms, and dangerous substances. The third cluster analyses the physical examination findings, which are already known to be possible markers of abuse (bruises, fractures, etc.).

**Table 1:** Screening indicators created in 2009 and used in clinical practice from 2010.

CLUSTER	ITEM
ANAMNESTIC DECLARATIONS OR INCONGRUENCES	Statement / accusation of abuse
	Statement / suspicion of domestic violence
	Statement / suspicion of sexual abuse
	A history that does not explain the cause of the injury or the detected pathologies, inconsistent medical history with the cause of the injuries, medical history not compatible with clinical objectivity detected
CARELESSNESS/NEGLECT	Exposure to the use of drugs
	Child in conditions of neglect
	Severe physical neglect (of care pathology)
	Obvious and serious lack of timely medical treatment and failure to respect medical care specifically recommended, which has adversely affected the health of a child
EVIDENT LESIONS AT THE PHYSICAL EXAMINATION	Unexplained bruising
	Unexplained burns or burns extended to more than 10% of the body surface, cigarette burns, burns on the genitals
	Evidence of traumatic injuries occurred at different times, especially on children aged 3 years or less
	Fractures without a coherent history. Fractures in children aged 3 years or less
	Drowning / violent mechanical asphyxia
	Precipitation

The screening activities must be reported in the health documentation for each individual subject: in first aid record upon arrival at the triage by the nurse and subsequently in medical assessment by physician, in case of hospitalization in the medical record in a specific session, dedicated to the "initial assessment" or in the case of outpatient visits in the medical evaluation.

**Fig. 1:**
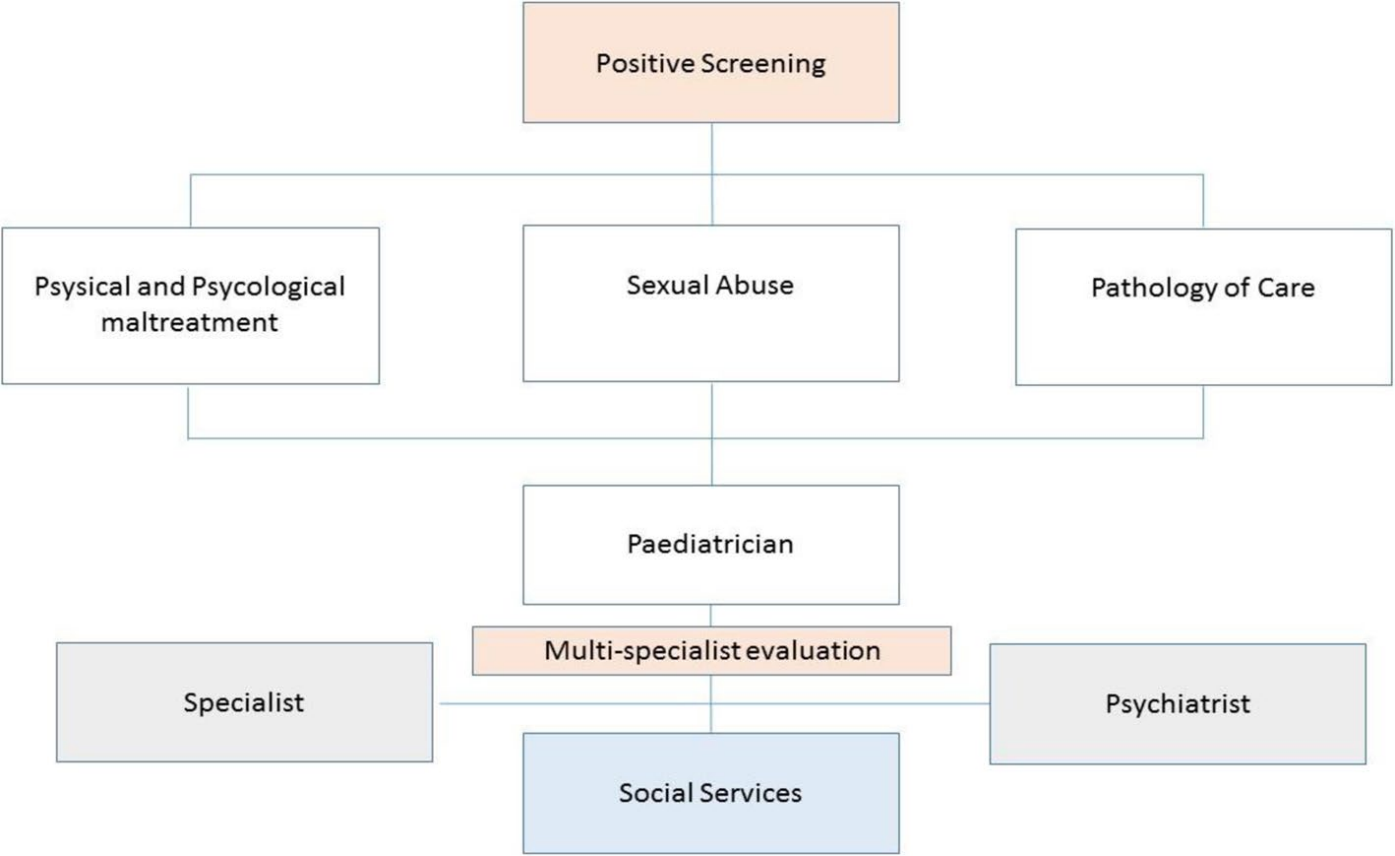
Flow-chart of the clinical pathways

If one or more items are found to be positive, a multidisciplinary team performs the clinical pathway. Each of the specialists, after completing the detection phase, will decide the type of diagnostic procedure and more appropriate treatment tailored for the specific patient. The collection of biological samples should be carried out as soon as possible (consistent with the clinical activities) directly in the emergency room. Clearly, in all subject we performed a complete history and physical examination, focussed on particularly oriented to the description of all presented lesions (injuries, abrasions, lacerations, bites, blood or other secretions, even outside of the genital or anal area). All diagnostic activities shall obey the principles of maximum response, minimal invasiveness and rigorous verification of possible differential diagnoses. Confirmation of suspicion leads to a referral to social services for child protection (Fig. 1 for detail). Moreover, once the clinical pathway was completed, the cases which were included into one of the three types of abuse were referred to the competent offices (Judicial Authority) Parents were informed about the subsequent effects of the report.

In 2013 the screening criteria were revised and modified to increase their specificity. Thereby the criteria relating to physical injuries have been modified to better investigate the anamnestic data and to better reconstruct the harmful dynamic, for example in an attempt to discriminate injuries likely related to common play activities and reduce the risk of false positive. So the items related to physical injuries were converting in “Evidence of multiple traumatic injuries that occurred at different times, not related to the usual games and sporting activities, particularly if under 3 years” and “Fractures under one year of life without coherent story” (Table 2). Stratification characteristics of all positive studied patients according to age, sex, and type of CAN was made.

**Table 2:** Screening indicators modified in 2013

CLUSTER	ITEM
ANAMNESTIC DECLARATIONS OR INCONGRUENCES	Inconsistencies medical history: a history that does not explain the causes of injuries or illnesses detected, inconsistent medical history about the cause of the injuries, medical history not compatible with clinical objectivity detected
	Statement (of the child carers) of a suspected abuse, domestic violence, harassment or sexual violence
CARELESSNESS/NEGLECT	Exposure to the use of drugs or substances
	History of previous abuse or domestic violence
	Child in conditions of neglect, or parents where parental responsibility is suspended
	Severe physical neglect causing pathological conditions of the minor (of care pathology)
	Obvious and serious lack of timely medical treatment and / or failure to comply with medical treatment specifically recommended, with prejudice to the health of a minor
EVIDENT LESIONS AT THE PHYSICAL EXAMINATION	Evidence of multiple traumatic injuries that occurred at different times, not related to the usual games and sporting activities, particularly if under 3 years
	Fractures under one year of life without coherent story
	Unexplained burns or extended to more than 10% of the body surface, cigarette burns, burns on the genitals
	Unexplained bruising
	Drowning / violent mechanical asphyxia
	Precipitation

## Results

**Fig. 2:**
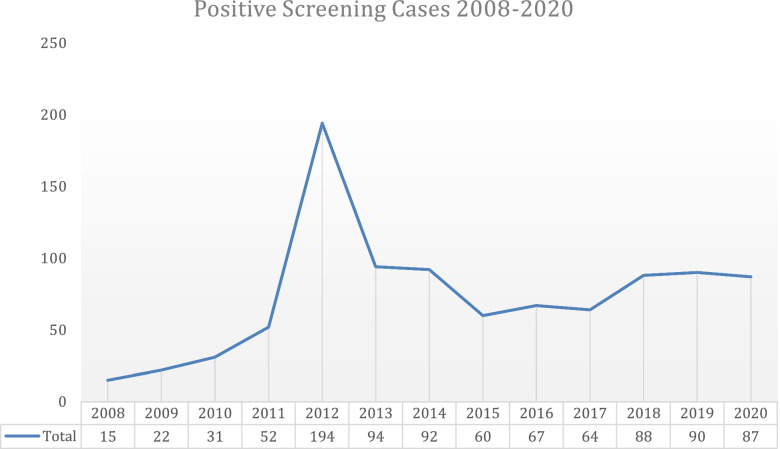
Total positive ED screening cases from 2010 to 2020

An estimation of positive screening patients, regarding the type of abuse suspected, and the number of accesses in ED was made. In 2008 and 2009, before the introduction of the screening, we reported a total of 15 and 22 suspected cases of abuse (Fig. 2). From 2010, year of introduction of screening composed of 14 items, we observed a gradual increment of suspected CAN. In fact, we reported 31 suspected cases in 2010, 52 cases in 2011 and in 2012 a total of 194 patients (Fig. 2).

In 2013, after three years of experience, the criteria were modified to increase specificity, leading to a median number of 82 suspected cases/year from 2013 to 2020. Positivity to one of the items allows the actuation of the investigating protocol named as clinical pathway. After the modification of the criteria in 2013 we observed a decrease in the amount of positive screening cases with an average of 82 patients/year (See Fig. 2 for numerical variations over the years in period study).

We did not find a reduction in the number of suspected cases even during the Covid-19 pandemic period. Patients characteristics according to age, sex, and type of CAN are shown in Table 3 and Table 4. Over the study period 956 subjects in total were studied (Table 3). According to age we stratified the population in 5 age groups. Four hundred and two infants (< 1 year) (377 patients) in which “neglet/carelesseness” group were more represented, followed by “maltreatment” and none classified as “sexual abuse”. Toddler (1- 3 years) group was constituted by 260 patients (28%): 220 as “neglet/carelesseness”, 28 “sexual abuse” and 20 “maltreatment”. In 4–6 years group (102 pts), reported sexual abuse in 44, “neglet/carelesseness” in 36 and “maltreatment” in 22. In 7–12 years group were 124 patients with 51 “neglet/carelesseness”, 39 “sexual abuse” and 34 “maltreatment” patients. Late adolescents (> 12 years) were 60 patients, subdivided in 24 “maltreatment”, 23 “sexual abuse” and 13 “neglet/carelesseness” (see Table 3 for detail). Considering all the patients studied, no difference was noted between males and females. Regarding the stratification for type of abuse, sex sexual abuse was most frequent in female while maltreatment in male (Table 4).

**Table 3:** Positive screening test performed per age group

Age	Number	Sexual Abuse	Neglect/Carelessness	Maltreatment
< 1 year	402 (42%)	0 (0%)	377 (54%)	25 (20%)
1–3 years	268 (28%)	28 (21%)	220 (32%)	20 (16%)
4–6 years	102 (11%)	44 (33%)	36 (5%)	22 (18%)
7–12 years	124 (13%)	39 (29%)	51 (7%)	34 (27%)
> 12 years	60 (6%)	23 (17%)	13 (2%)	24 (19%)
Total	956	134	697	125

**Table 4:** Positive screening test according to sex and type of child abuse and neglect

	**Total**	**Male**	**Female**
Sexual Abuse	134 (14%)	35 (7%)	99 (21%)
Neglect/Carelessness	697 (73%)	378 (78%)	319 (68%)
Maltreatment	125 (13%)	74 (15%)	51 (11%)
Total	956	487	469

## Discussion

Our experience suggests that a screening tool is essential and productive for the early recognition of victims of suspected abuse through a standardized method, such as a clinical pathway, allowed reaching the diagnosis in a more accurate and precise manner, especially in emergency setting, decreasing the portion of abuse victims who remain underdiagnosed. In our study, the introduction of screening tools led to a diagnostic improvement due to the increase of sensitivity of health workers to the phenomenon and standardization of diagnostic path. In our hospital after the modification of the screening criteria, after 3 years of experience (Table 2), we noticed a conspicuous increase in the ability of interception. Another improvement was made to highlight at first point the anamnestic declarations or incongruences.

One of the main difficulties, when comparing between the incidences detected in different countries, is that the phenomenon of the abuse is not unequivocally defined, but it is experienced and recognised differently depending on the social and cultural background.

Carelessness and neglect represent the most susceptible situations for different cultural interpretations, changing meaning not only from place to place, but also in different times.

Therefore, it may be important to compare literature data from countries with similar cultural collective values to truly understand the dynamics under every founding.

Various researchers have therefore taken steps to codify “indicators of abuse” and to measure their sensitivity and specificity. Following there are some examples of how some of the indicators have been used and tested, and the success they have reported. The introduction of a checklist of 10 questions administered by ED nurses to Canadian patients with trauma was able to increase the ability of recognition from 0.86% to 1.13% (95% CI 0.72 OR 1:32 to 2:40). This increase in sensitivity, however, was not statistically significant and was not able to effectively intensify the capability of the screening [19]. In a UK study, using certain indicators in clinical practice with a prospective study of 2345 patients, was demonstrated that using a screening method increased the recognition ability of abused victims [20]. The indicators were: whether the patients had already been in the ED, if there was an inconsistent medical history, physical examination and clinical history not consistent with each other, if there had been a delay in bringing the child to the ED compared to the trauma and if there were skull fractures in children below one year of age [20]. A next study showed that the use of 8 items, from nurses at the ED, increased awareness and vigilance of health professionals and proposed a flow chart on how to behave in case of positive indicators [14]. Also in the UK, two studiesreviewed and evaluated the sensitivity of screening without finding a test with high sensitivity to physical abuse and the only item found to aid clinicians in assessing the abuse victim was the "type" of injury [21, 22].

A Netherlands study brought to light that the use of 9 questions as indicators of abuse could improve the recognition of cases on 220 suspected cases. [23] A Dutch study showed that 6 simple questions could get a 80% sensitivity and 98% specificity for abuse; in fact, on a sample of 420 children with positive screening, 44 had been confirmed; on 17,855 children with negative screening, only 11 were instead victims of abuse [24]. The indicators applied were if the history was consistent, if there was a delay in seeking for medical help, if the onset of the injury fit with the development level of the child, if the behaviour of the child and his/her careers were appropriate, in the head-to-toe examination was in accordance with the history and if other signals could make the nurse doubt on the safety of the child or other family members.

A previous work suggested a checklist consisting of 9 questions, defined SPUTOVAMO, associated to a combination of both a complete physical inspection of every child (called ‘top–toe’ inspection) presenting at the ED, and a system of standard referral of all children from parents who attend the ED for intoxication, severe psychiatric disorders or with injuries due to intimate partner violence. This was presented by the Authors as the most promising procedure for the early diagnosis of CAN in the emergency setting [25]. Most recently the same group confirmed that combining screening test (SPUTOVAMO screening checklist, complete physical examination and their combination) significantly increases the number of test positives and led to more child abuse cases being detected than using either method on its own [26]. It would be desirable for each emergency department to have its own screening tool.

Susceptibility to maltreatment decreases with age placing younger children at the greatest risk for abuse and neglect [27]. In agreement, in our study we observed that visits for maltreatment most often involved children who were significantly younger (Table 3). This is likely a result of the limited verbal aptitude of young children to articulate their maltreatment experience, as observed in other study [27]. The clinical practice of asking parents or caregivers for information about the event, especially valid in case of uncertainty about the circumstances of the "symptoms of possible maltreatment", represents a limit, especially in young children, considering that parents and caregivers are the possible main perpetrators of the maltreatment, as already reported by other Authors [27, 28]. The application of a clinical pathway and the possibility of having a multidisciplinary team in the hospital may certainly contribute to identify maltreatment in children with suspicious or unexplainable injuries, increasing objectivity and standardization, as happened in our study.

In agreement with other previous studies [27, 29], our study identified a difference in the distribution of child sex maltreatment (Table 4); specifically, females are more likely to experience sexual abuse, while males are more likely to experience physical abuse.

To screen the totality of the patients that access to the hospital is necessary to early recognize suspected CAN. If one or more items are found to be positive it is important for us that a multidisciplinary team performs the clinical pathway. Our clinical pathways provide different approaches depending on which item results positive after the screening process and the different specialists involved in the clinical assessment depending on the type of considered CAN category (sexual abuse, neglect/carelessness, maltreatment) (Table 3). In these terms, the importance of an already codified pathway helps bringing together the different specialties in a more productive and efficient collaboration (Fig. 1).

### Limitations

Mostly because of retrospective design and study setting, our study suffers at least two limitations that could affect our conclusion. First, data have been extracted from ED medical records. Albeit many patients received multidisciplinary consultations in the ED, accuracy of CAN assessment may have been partially limited by the emergency setting. Moreover, several patients (especially non-hospitalized patients) did not receive a definite diagnosis for their disturbance, reflecting the lack of information about the diagnostic work-up after ED discharge. Second, the generability may be limited because this study was conducted in a ED of a pediatric tertiary hospital with all specialties, and in Italy as in other country [e.g., United States [30] many children are conducted directly in general ED, many of which may not specifically access to pediatric multidisciplinary team, comprising social work team, specialized for CAN.

## Conclusion

Our experience suggests that a screening tool is essential and productive for the early recognition of victims of abuse. Furthermore, the in-deep analysis of suspected cases through a standardized method, such as the clinical pathway, allowed reaching the diagnosis in a more accurate and precise manner. The possibility to further implement the sensitivity of these screening methods remains a major goal for healthcare professionals in the ED, with the hope of decreasing the portion of abuse victims who remain undiagnosed and preventing the long-term consequences of suffering children.

A desirable future step would be to share these indicators with non-paediatric hospitals,that do not have the same experience in recognizing cases of abuse. It remains still unclear when to consider the notification of neglect appropriate: as a matter of fact, different social backgrounds can influence the seriousness of the neglect. A clinical pathway focusing on early involvement of a multidisciplinary pediatric team and social work team in the early detection of child abuse are advocated.

## Data Availability

Availability of data and materials at Bambino Gesù Children Hospital.

## Abbreviations

CAN: Child Abuse and Neglect
ED: Emergency Department
